# Meaning in life, emptiness and depression among adolescents: a cross-sectional study

**DOI:** 10.3389/frcha.2026.1836277

**Published:** 2026-06-17

**Authors:** Yue Sun, Yiting Zhao, Zhiyuan Xun, Jingjie Yu

**Affiliations:** 1 Department of Psychiatry and Psychology, Tianjin Union Medical Center, The First Affiliated Hospital of Nankai University, Tianjin, China; 2 Children's Hospital, Tianjin University, Tianjin, China; 3 Tianjin Anding Hospital, Tianjin, China

**Keywords:** adolescent depression, emptiness, mental health, presence of meaning, searching for meaning

## Abstract

**Objective:**

To investigate the relationship between meaning in life, emptiness and depression among adolescents.

**Methods:**

A cross-sectional study was conducted using convenience sampling at a Senior High School in China, in May 2022. Validated questionnaires, including Center for Epidemiological Survey Depression Scale (CES-D), Revised Meaning in Life Questionnaire (RMLQ), and Emptiness Questionnaire (EQ), were administered to senior high school students. Binary logistic regression was employed to analyze associations between depression and independent variables.

**Results:**

A total of 2230 senior high school students were included, among whom 726 (32.6%) were classified as depressed (DEP) and 1504 (67.4%) as non-depressed (non-DEP). Compared with non-DEP, the DEP group had a significantly higher proportion of females (49.7% vs. 39.9%; *χ*^2^ = 19.30, p < 0.001). Moreover, the DEP group reported higher levels of emptiness (39.59 ± 7.63 vs. 37.34 ± 7.62, p < 0.001), lower presence of meaning (21.56 ± 6.32 vs. 26.97 ± 6.19, p < 0.001), and lower searching for meaning (24.85 ± 5.82 vs. 26.18 ± 7.05, p < 0.001). Correlation analyses revealed that depressive symptoms were positively associated with emptiness (r = 0.16) and negatively with presence of meaning (r = −0.46) and searching for meaning (r = −0.12) (all p < 0.001). In hierarchical logistic regression, emptiness (OR = 1.03, p < 0.001) and presence of meaning (OR = 0.88, p < 0.001) were significant predictors of depression, whereas searching for meaning was not (*p* = 0.08). The combined prediction model (emptiness, presence of meaning, and demographics) showed an AUC of 0.74 (95% CI: 0.72-0.76), indicating acceptable discriminative ability.

**Conclusion:**

In this cross-sectional sample, emptiness and limited presence of meaning in life were concurrently associated with depression status among high school students. Although directionality cannot be determined, these findings suggest that interventions targeting these constructs may warrant evaluation in future longitudinal and experimental research, and their combination shows potential for school-based screening.

## 1 Introduction

Adolescence constitutes a critical developmental window marked by rapid reorganization of psychological, social, and existential identity structures (1). Within this period, the sense of meaning in life has emerged as a core indicator of mental health and adaptive functioning (2). According to Steger et al., meaning in life comprises two related yet conceptually distinct dimensions: the presence of meaning (PM), reflecting the extent to which individuals perceive their lives as meaningful, and the search for meaning (SM), reflecting the motivational drive to discover or deepen one's sense of purpose (3). While PM consistently predicts adaptive psychological outcomes across developmental stages, the implications of SM during adolescence remain theoretically contested (4–6). Some evidence suggests that SM, in the absence of corresponding gains in PM, may reflect existential distress rather than purposeful exploration, underscoring the importance of examining these two dimensions independently rather than as a unified construct (7, 8).

Parallel to the construct of meaning in life, the experience of emptiness has gained increasing clinical recognition among youth populations (9). Conceptualized as a pervasive inner sense of void, disconnection, and purposelessness, emptiness is phenomenologically distinct from sadness or anxiety in that it is characterized not by the presence of distressing affect, but by its relative absence (10). This experiential state has been theoretically linked to emotional dysregulation and multiple forms of psychopathology, yet rigorous empirical investigation of its correlates within non-clinical adolescent samples remains sparse. Complementing these existential dimensions, life satisfaction, as the cognitive-evaluative component of subjective well-being, has been consistently identified as a protective factor against adolescent depression (11). Whereas emptiness and meaning in life capture the existential and motivational textures of adolescent experience, life satisfaction reflects individuals’ deliberate cognitive appraisal of their overall life circumstances. Together, these constructs may represent complementary pathways through which psychological vulnerability to depression is shaped.

Depression ranks among the most prevalent and impairing mental health conditions in adolescence, with its global burden continuing to escalate (12). Established risk factors, including genetic predisposition, family conflict, and academic stress, have been extensively documented (13, 14). Nevertheless, existential vulnerabilities, such as a diminished sense of meaning or pervasive feelings of inner emptiness, may represent underexplored yet clinically significant mechanisms in the onset and maintenance of adolescent depression. Understanding how these existential constructs relate to and are associated with depressive symptomatology may inform novel avenues for early identification and targeted intervention.

Although longitudinal designs would be optimal for establishing directionality, the present cross-sectional study constitutes a necessary preliminary step by mapping the concurrent associations among these constructs and generating effect-size estimates to inform the statistical power of future prospective research. To the best of our knowledge, no prior study has simultaneously examined the independent and combined contributions of PM, SM, emptiness, and life satisfaction to depression status within a large, non-clinical adolescent sample. The present study addresses this gap by integrating existential, motivational, and evaluative dimensions of well-being into a unified predictive model, thereby providing an empirically grounded foundation for subsequent longitudinal and intervention research.

It is also important to contextualize this study temporally. Data were collected in May 2022, a period during which Chinese adolescents were still experiencing the lingering effects of the COVID-19 pandemic, including intermittent lockdowns, prolonged social restrictions, and disrupted educational routines (15). Emerging evidence indicates that mental health symptoms fluctuated across pandemic stages, with recurrence-stage restrictions associated with elevated depression and anxiety in general and youth populations (16). Consequently, the levels of emptiness, meaning disruption, and depressive symptoms observed here may have been partially shaped by these unprecedented contextual stressors.

Based on the foregoing review, the present study tested the following hypotheses: (1) Significant demographic differences would exist between DEP and non-DEP groups, with female students being disproportionately represented in the DEP group; (2) Depressive symptoms would be positively associated with emptiness and negatively associated with PM and life satisfaction; given equivocal evidence in the literature, the association between SM and depressive symptoms was treated as exploratory; (3) Emptiness would be positively associated with depression risk, and PM would be negatively associated with depression risk in a multivariate logistic regression model; the independent contributions of SM and life satisfaction were treated as exploratory; (4) The combined predictive model would demonstrate acceptable discriminative accuracy, as indexed by an area under the receiver operating characteristic curve (AUC) exceeding 0.70.

To the best of our knowledge, this is the first study to concurrently test the independent contributions of existential emptiness, presence of meaning, and search for meaning to adolescent depression in a large school-based sample, thereby providing an initial evidence base for integrating existential constructs into youth mental-health screening.

## 2 Methods

### 2.1 Participants

Participants were senior high school students recruited from a single school in China via convenience sampling. Electronic questionnaires were distributed to all enrolled students through the Questionnaire Star platform. Participants were eligible for inclusion if they were: (1) currently enrolled as full-time students at the target senior high school; (2) aged between 14 and 18 years; and (3) willing to provide voluntary participation. Participants were excluded if they: (1) completed the questionnaire in less than 10 minutes; (2) failed two or more embedded attention-check items; or (3) had a self-reported current diagnosis of a psychiatric disorder or were receiving ongoing psychological or pharmacological treatment at the time of the survey.

All participants were informed of the voluntary and anonymous nature of the study prior to completing the questionnaire and were explicitly advised that they could withdraw at any time without consequence. Given that participants were minors, passive parental consent was obtained through prior notification to school administration. The study protocol was approved by the Ethics Committee of Tianjin Anding Hospital (Approval No.: 2020-15).

### 2.2 Measures

#### 2.2.1 Demographic questionnaire

A self-administered demographic questionnaire was developed to collect information on participants’ gender, grade level, boarding status, and only-child status.

#### 2.2.2 The center for epidemiological studies depression scale (CES-D)

Depressive symptoms were assessed using the CES-D (17), a 20-item self-report instrument measuring symptom frequency over the past week on a 4-point Likert scale (0 = rarely or never to 3 = most of the time or constantly). Total scores range from 0 to 60. Among Chinese high school students, confirmatory factor analysis supported the four-factor structure with acceptable fit (*χ*^2^/df = 4.344, CFI = 0.915, NNFI = 0.900, RMSEA = 0.057) (18). In the present sample, Cronbach's *α* was 0.893. A cutoff score of ≥16 was applied to classify participants into the depression group (DEP) and non-depression group (non-DEP).

#### 2.2.3 Revised meaning in life questionnaire

The sense of meaning in life was assessed using the RMLQ (19), a 10-item instrument comprising two 5-item subscales: Presence of Meaning (PM) and Search for Meaning (SM). Items were rated on a 7-point Likert scale (1 = strongly disagree to 7 = strongly agree). Higher subscale scores indicate greater presence of or greater active search for meaning in life, respectively. The Chinese version has demonstrated satisfactory psychometric properties in previous studies, with confirmatory factor analysis supporting its original two-factor structure (20): *χ*^2^/df = 2.31, CFI = 0.96, IFI = 0.96, RMSEA = 0.077. In the present sample, the Cronbach's *α* was 0.890 for PM, 0.870 for SM, and 0.865 for the total scale.

#### 2.2.4 Emptiness questionnaire (EQ)

An 18-item scale assessing emptiness across four dimensions: sense of value, freedom of will, negative emotions, and negative behaviors. Items are rated on a 5-point Likert scale (1 = strongly disagree to 5 = strongly agree), with higher total scores indicating higher levels of experienced emptiness. In the original validation study (21), the EQ demonstrated strong psychometric properties, including Cronbach's *α* = 0.909 and confirmatory factor analysis fit indices of *χ*^2^/df = 2.792, CFI = 0.998, TLI = 0.987, and RMSEA = 0.053. In the present sample, Cronbach's *α* was 0.831, indicating acceptable internal consistency.

#### 2.2.5 Satisfaction with life scale (SWLS)

Life satisfaction was assessed using the SWLS (22), a 5-item scale measuring global cognitive judgments of one's life. Items are rated on a 7-point Likert scale (1 = strongly disagree to 7 = strongly agree), with higher total scores indicating greater life satisfaction. The unidimensional structure of the SWLS has been previously validated in Japanese samples, with satisfactory model fit indices (*χ*^2^/df = 3.438， CFI = 0.990， RMSEA = 0.064) (23). In the present sample, Cronbach's *α* was 0.881.

### 2.3 Statistical analysis

All statistical analyses were conducted using SPSS version 25.0 (IBM Corp., Armonk, NY, USA). Continuous variables are reported as mean ± standard deviation (SD), and categorical variables as frequency and percentage. Between-group comparisons of categorical variables were performed using Pearson's chi-square test. Prior to inferential analyses of continuous variables, normality was assessed using the Kolmogorov–Smirnov test. Although statistically significant deviations from normality were detected for several variables—as is common in large samples due to the high statistical power of normality tests—independent-samples t-tests were nonetheless employed for between-group comparisons of continuous variables, given their well-documented robustness to mild violations of normality in large samples. Pearson product-moment correlations were used to examine bivariate associations among continuous variables.

Binary logistic regression was conducted to examine the independent contributions of PM, SM, emptiness, and life satisfaction to depression group membership (DEP vs. non-DEP). Demographic variables that demonstrated significant between-group differences in preliminary analyses were entered in the first block as covariates, followed by the four psychological predictor variables in the second block, using the forced-entry method. Odds ratios (ORs) and 95% confidence intervals (CIs) are reported for all predictors. The discriminative accuracy of the full regression model was evaluated via receiver operating characteristic (ROC) curve analysis, with area under the curve (AUC) as the primary index of model performance. An AUC of 0.70 was adopted as the minimum threshold for acceptable discrimination, consistent with established conventions cite. The optimal cutoff point was determined using the Youden index (sensitivity + specificity−1). Multicollinearity among predictors was assessed using tolerance values and variance inflation factors (VIF); tolerance >0.1 and VIF <10 were used as thresholds for acceptable collinearity, with actual values reported in the results. All tests were two-tailed, with a significance threshold of *α* = 0.05.

To assess the potential common method variance (CMV), Harman’s single-factor test was performed via exploratory factor analysis on all scale items.

## 3 Results

### 3.1 Demographic and psychological characteristic of participants

A total of 2230 senior-high-school students (mean age = 16.62 ± 0.97 years) were included in the analyses. Of them, 726 (32.6%) met the cut-off criteria for the DEP and 1 504 (67.4%) were assigned to the non-DEP. Descriptive statistics for the whole sample and for each group are summarized in Table 1.

**Table 1:** Socio-demographic and psychological characteristics in senior high school students with and without depression.

Variables	Total samples (*n* = 2230)	DEP (*n* = 726)	non-DEP (*n* = 1504)	*t/χ^2^*	*P* value
Age	16.62 ± 0.97	16.60 ± 1.01	16.63 ± 0.95	0.75	0.45
CES-D	13.03 ± 9.37	24.05 ± 7.30	7.72 ± 4.18	66.98	<0.001
Emptiness	38.07 ± 7.70	39.59 ± 7.63	37.34 ± 7.62	6.54	<0.001
PM	25.20 ± 6.73	21.56 ± 6.32	26.97 ± 6.19	−19.22	<0.001
SM	25.74 ± 6.70	24.85 ± 5.82	26.18 ± 7.05	−4.42	<0.001
Life satisfaction	22.84 ± 6.45	22.87 ± 6.39	22.83 ± 6.49	0.15	0.88
Gender (%)					
Male	1269 (56.9)	365 (50.3)	904 (60.1)	19.30	<0.001
Female	961 (43.1)	361 (49.7)	600 (39.9)		
grade (%)					
Senior-1	777 (34.8)	263 (36.2)	514 (34.2)	1.41	0.50
Senior-2	873 (39.1)	272 (37.5)	601 (40.0)		
Senior-3	580 (26.0)	191 (26.3)	389 (25.9)		
boarding status (%)					
Yes	1932 (86.6)	633 (87.2)	1299 (86.4)	0.29	0.59
No	298 (13.4)	93 (12.8)	205 (13.6)		
only-child status (%)					
Yes	260 (11.7)	84 (11.6)	176 (11.7)	0.01	0.93
No	1970 (88.3)	642 (88.4)	1328 (88.3)		

CES-D, The Center for Epidemiological Survey Depression Scale; PM, Presence of Meaning; SM, Searching for Meaning.

Overall, 43.1% of the participants were female, 86.6% were boarders, and 11.7% were single children. Grade distribution was 34.8% senior-1, 39.1% senior-2 and 26.0% senior-3. Mean scores on the study variables were as follows: CES-D 13.03 ± 9.37, emptiness 38.07 ± 7.70, PM 25.20 ± 6.73, SM 25.74 ± 6.70, and life satisfaction 22.84 ± 6.45.

### 3.2 Comparison of the characteristics

Table 1 compared the differences in personal characteristics between DEP (*n* = 726) and non-DEP (*n* = 1504). No significant difference was observed between groups in regard to age, grade, boarding status, only-child status, and life satisfaction (all *P* > 0.05). However, there was a significant difference in gender between DEP and non-DEP (*χ^2^* = 19.30, *df* = 1, *P* < 0.001), with a higher proportion of females in the DEP (49.7%) compared to the non-DEP (39.9%). Furthermore, significant differences were observed between groups in the score of emptiness (39.59 ± 7.63 versus 37.34 ± 7.62, *t* = 6.54, *P* < 0.001), the score of PM (21.56 ± 6.32 versus 26.97 ± 6.19, *t* = −19.22, *P* < 0.001), and the score of SM (24.85 ± 5.82 versus 26.18 ± 7.05, *t* = −4.42, *P* < 0.001).

### 3.3 Variables associated with depression in senior high school students

Correlation analysis (Table 2) revealed significant correlations between CES-D total score and the following parameters: emptiness score (*r* = 0.16, *P* < 0.001), PM score (*r* = −0.46, *P* < 0.001) and SM score (*r* = −0.12, *P* < 0.001). Life satisfaction score was not significantly correlated with CES-D total score (*r* = 0.01, *P* = 0.67). Given that life satisfaction demonstrated no significant bivariate correlation with depression severity and was included as an exploratory rather than theoretically mandated predictor, it was excluded from the hierarchical logistic regression model to preserve parsimony and avoid overfitting. Consequently, the final model included only emptiness, PM, and SM as the psychological predictors, alongside the demographic covariates.

**Table 2:** Inter-relationship between clinical variables in senior high school students.

variables	CES-D	Emptiness	Presence of meaning	Searching of meaning	Life satisfaction
CES-D	1				
Emptiness	0.162^a^	1			
PM	−0.457^a^	−0.166^a^	1		
SM	−0.117^a^	−0.035	0.338^a^	1	
Life satisfaction	0.009	−0.005	−0.002	−0.027	1

CES-D, The Center for Epidemiological Survey Depression Scale; PM, Presence of Meaning; SM, Searching for Meaning.

a *P*＜0.01.

We performed a hierarchical logistic regression, entering demographic variables in the first block and continuous variables (emptiness, PM, SM) in the second block. The final model (Table 3) explained 20.7% of the variance (Nagelkerke *R*^2^). In this concurrent model, emptiness (OR = 1.03, 95% CI = 1.01–1.04, *P* < 0.001) and PM (OR = 0.88, 95% CI = 0.86–0.89, *P* < 0.001) were significantly associated with depression group membership, whereas SM (OR = 1.02, 95% CI = 0.99–1.03, *P* = 0.08) was not independently associated with group membership.

**Table 3:** Hierarchical logistic regression for senior high school students.

Predictor	Model 1	Model 2
	OR [95% CI]	OR [95% CI]
Step 1: Demographic Variables		
Gender	1.50^a^[1.25,1.79]	1.34^b^[1.11,1.64]
Grade	0.98[0.87,1.10]	1.02[0.90,1.15]
Boarding status	1.07[0.82,1.39]	1.00[0.75,1.34]
Single-child status		0.99[0.73,1.35]
Step 2: Psychological Variables		
Emptiness		1.03^a^[1.01,1.04]
Presence of meaning		0.88^a^[0.86,0.89]
Searching of meaning		1.02[0.99,1.03]
Model fit		
Nagelkerke R^2^	0.012	0.21
Model χ^2^	4.39	11.61

a *P* < 0.001

b *P* < 0.01

**Figure 1:**
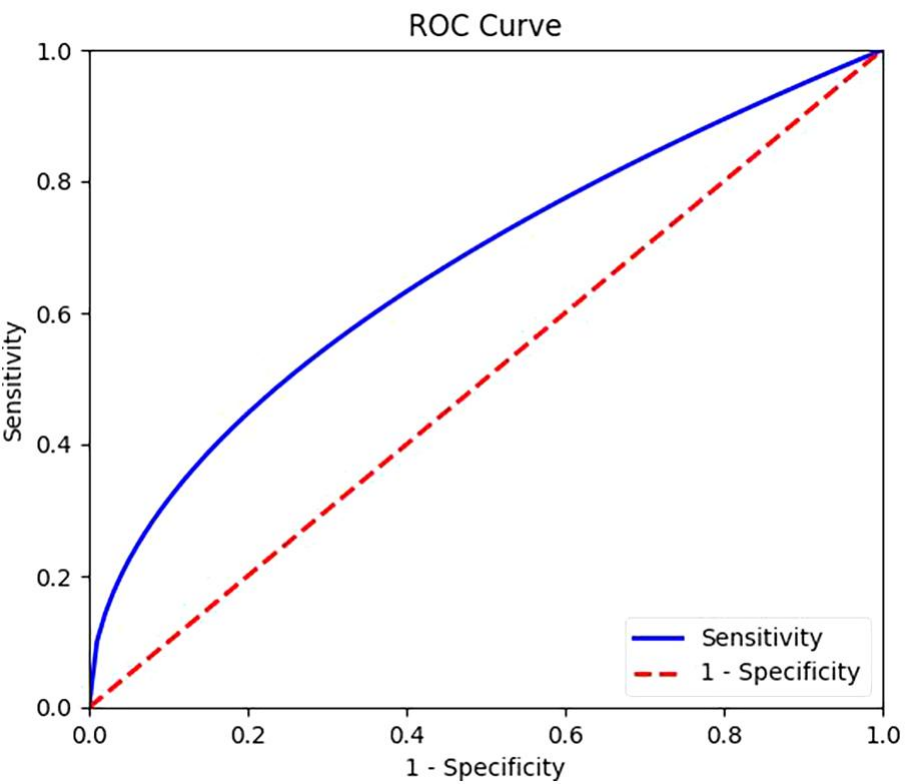
ROC curve of the combined model (gender, emptiness, and PM) for predicting depression.

We conducted ROC analysis for the combined model (gender, emptiness, PM). The area under the curve (AUC) for the combined model was 0.74 (95% CI: 0.72-0.76, *P* < 0.001), exceeding the prespecified threshold of 0.70 for acceptable discrimination. The optimal cutoff probability was 0.357, determined by the maximum Youden index (0.348), yielding a sensitivity of 64.3% and a specificity of 70.5%. The ROC curve is shown (20) in Figure 1.

### 3.4 Common method variance assessment

To examine whether CMV posed a substantial threat, we performed Harman's single-factor test via exploratory factor analysis on all scale items. Six factors with eigenvalues exceeding 1.0 were extracted, and the first unrotated factor accounted for 27.92% of the total variance. This value is below the 40% threshold sometimes used as a rough heuristic for CMV concerns, though we acknowledge that this test is widely regarded as insensitive and cannot definitively rule out method bias.

## 4 Discussion

### 4.1 Core findings in brief

This cross-sectional study examined the concurrent associations among depressive symptoms, emptiness, and dimensions of meaning in life in a large sample of Chinese senior high school students. In support of the hypotheses, both emptiness and presence of meaning were associated with depression. Higher emptiness was linked to increased odds of depression, whereas higher PM was linked to reduced odds. Searching for meaning, despite a negative bivariate correlation, did not emerge as a unique predictor in the multivariate model. Female students were overrepresented in the DEP group, corroborating well-established gender disparities in adolescent internalizing symptoms. The combined model (gender, emptiness, and PM) achieved an AUC of 0.74, suggesting acceptable discriminative accuracy for distinguishing DEP from non-DEP students in this concurrent, school-based context. Because the data were collected at a single time point, all observed associations are inherently correlational and should not be interpreted as causal, directional, or temporal risk factors.

### 4.2 Prevalence, gender differences, and the COVID-19 context

The prevalence of elevated depressive symptoms (32.6%) aligns with recent meta-analytic estimates among adolescents (24, 25) and underscores the substantial mental health burden in this population. The higher proportion of females in the DEP group (49.7% vs. 39.9%) is consistent with the well-established female preponderance in adolescent depression (26), a disparity thought to arise from the interplay of biological (e.g., hormonal changes), psychological (e.g., heightened rumination), and sociocultural (e.g., body-image and interpersonal pressures) factors (27).

Furthermore, data were collected in May 2022 under stringent COVID-19 restrictions (campus lockdowns, social isolation) (15, 16). which may have elevated depression and emptiness levels; caution is therefore warranted when generalizing to non-pandemic cohorts. Beyond these demographic and contextual factors, the focal psychological constructs revealed distinct association profiles.

### 4.3 Emptiness as a distinct existential correlate

Consistent with prior research, emptiness emerged as a significant concurrent correlate of depression status, supporting its conceptualization as a transdiagnostic marker within the internalizing spectrum (28, 29). Emptiness encompasses numbness, disconnection, and purposelessness (30, 31)—features that phenomenologically overlap with depressive disturbances such as anhedonia and worthlessness, yet are distinct in their characterization as an affective absence rather than active distress (10). Although our cross-sectional design precludes etiological tests, developmental research has posited that early relational trauma may manifest as chronic emptiness, heightening vulnerability to depression under stress (32). Clinically, assessing emptiness may help identify at-risk adolescents who do not yet meet full symptomatic criteria, and interventions fostering social connectedness could potentially buffer its deleterious effects, pending experimental validation.

### 4.4 Presence of meaning as a protective correlate

In contrast to emptiness, higher PM was robustly associated with lower depression scores. This finding aligns with meta-analytic evidence demonstrating a stable negative association between PM and psychological distress across diverse populations (33). According to self-determination theory, meaning fulfills basic psychological needs (autonomy, competence, relatedness), which may promote well-being and attenuate vulnerability to depression (34). Among Chinese adolescents, PM has been shown to covary with lower depression via diminished alienation and enhanced life satisfaction (35). Moreover, PM has been identified as a mediator of the link between SM and mental health, indicating that merely seeking meaning is insufficient without the cognitive affirmation of having found it (36). From a clinical perspective, interventions that strengthen adolescents’ sense of purpose (e.g., values clarification, goal-setting, prosocial engagement) represent a promising avenue for depression prevention, pending rigorous evaluation in longitudinal and experimental designs.

### 4.5 Searching for meaning: bivariate protection but no unique contribution

Although SM showed a significant negative bivariate correlation with depressive symptoms, it was not independently associated with depression status in the multivariate model. This aligns with theoretical distinctions between the two dimensions of meaning in life (37, 38): SM reflects a motivational process that may function independently of, or even inversely to, the cognitive resource of PM. Person-oriented studies demonstrate that SM is associated with positive outcomes only when accompanied by high PM; in its absence, SM is linked to poorer adjustment (8, 39). Indeed, Germani et al. (40) found that among early adolescents, family allocentrism was significantly related to PM but not to SM, suggesting that SM may operate through different mechanisms. For adolescents navigating identity formation, SM may represent normative exploration, yet without the cognitive anchor of PM, this search may fail to confer psychological benefits (41). Chen et al. (36) further demonstrated that SM predicted depressive symptoms only in the context of high-impact life events, indicating its context-dependent nature.

The discrepancy between bivariate and multivariate findings can be attributed to the moderate positive correlation between PM and SM (*r* = 0.338, *P* < 0.001). The apparent protective effect of SM in bivariate analysis likely reflects shared variance with PM; once PM is controlled, SM no longer contributes unique predictive variance. This pattern is consistent with a suppression effect, wherein SM may be adaptive when PM is high but maladaptive or neutral when PM is low. Thus, the null multivariate finding does not negate SM's theoretical importance; rather, it underscores that the benefits of existential search are contingent upon the concurrent experience of meaning. Clinically, interventions should prioritize consolidating adolescents’ existing sense of meaning rather than solely encouraging its pursuit.

### 4.6 Predictive model performance and the exclusion of life satisfaction

Moving from individual predictors to model-level evaluation, the combined model (gender, emptiness, PM) demonstrated acceptable discriminative accuracy (AUC = 0.74), suggesting potential, pending prospective validation, as a first-stage screening index for adolescent depression in school settings. Nevertheless, the unexplained variance (Nagelkerke R^2^ = 0.21) highlights the need for future studies to incorporate additional psychosocial factors, such as family environment, academic stress, and peer relationships.

Turning to the rationale for excluding life satisfaction, contrary to our exploratory expectation in Hypothesis 2, life satisfaction demonstrated no significant bivariate correlation with depression severity in this sample (*r* = 0.01, *P* = 0.67). Its exclusion from the final hierarchical regression model was therefore justified on grounds of parsimony: retaining a non-significant exploratory variable would have increased model complexity and risk of overfitting without explanatory gain. Although life satisfaction is theoretically relevant as a cognitive-evaluative component of subjective well-being and has been identified as a protective factor in prior adolescent depression research (11), this null finding suggests that, when more proximal existential variables (emptiness and PM) are considered, global appraisals of life circumstances may not independently distinguish depressed from non-depressed adolescents. It is plausible that life satisfaction operates as a distal outcome or mediator of adolescent depression, shaped in part by the more proximal existential states of emptiness and meaning, rather than as a direct predictor competing with them for shared variance. Future research should formally test whether life satisfaction mediates or moderates pathways linking existential constructs to depression over time.

### 4.7 Theoretical and clinical implications

Collectively, these findings advance a “having versus seeking” framework, demonstrating that emptiness and presence of meaning are distinct constructs with opposing concurrent associations, and that attained meaning—not active search—constitutes the primary protective resource against adolescent depression. School-based screening may benefit from incorporating brief measures of emptiness and PM alongside conventional symptom checklists to detect at-risk students earlier. Prevention efforts should prioritize reducing emptiness (e.g., fostering social connection) and consolidating existing sources of meaning (e.g., values clarification, prosocial engagement) over indiscriminately encouraging adolescents to “find their purpose”. Comprehensive assessment batteries integrating existential, psychological, and social domains are recommended to capture the multifaceted nature of youth depression. Future research should also consider incorporating validated, developmentally sensitive measures of adolescent depression that capture symptom heterogeneity and subthreshold presentations (42).

### 4.8 Limitations and future directions

Several limitations should be considered. First, the cross-sectional design precludes causal or directional inferences; the observed associations among emptiness, meaning, and depression may equally reflect reverse causality or unmeasured confounding (e.g., chronic stress, trauma history). Second, the convenience sample from a single urban high school—predominantly boarding students (86.6%)—limits generalizability to vocational schools, rural populations, non-boarding adolescents, youth outside formal academic contexts, or post-pandemic cohorts, and multi-site probability sampling is therefore needed. Third, all constructs were assessed via self-report in a single session. Although Harman's single-factor test indicated no severe CMV (first factor = 27.9%), this method is widely considered insensitive and cannot rule out method bias， which increases the susceptibility to CMV. Although we conducted Harman's single-factor test as a preliminary diagnostic, this procedure is widely considered insensitive and cannot statistically rule out method bias. Consequently, CMV cannot be fully excluded and remains a plausible threat to the validity of the observed associations. Future studies should employ multi-method approaches (e.g., clinical interviews, informant reports, ecological momentary assessment). Fourth, data were collected in May 2022 during stringent COVID-19 restrictions, which may have elevated depression and emptiness levels; future research should examine whether these associations persist in non-pandemic contexts and incorporate unmeasured confounders (e.g., family dysfunction, academic burnout, peer victimization) in longitudinal or experimental designs.

## 5 Conclusion

In this cross-sectional school-based sample, higher emptiness was concurrently associated with depression, whereas higher presence of meaning was concurrently associated with lower odds of depression. Because temporal precedence cannot be established, these relationships should be interpreted as correlational. Searching for meaning was not independently associated with depression status once presence of meaning was accounted for. Gender differences were evident, and the combined model demonstrated acceptable discriminative accuracy (AUC = 0.74). These divergent patterns for emptiness, presence of meaning, and search for meaning underscore the value of distinguishing existential risk markers from protective cognitive resources in adolescent depression research. These findings highlight the importance of attending to existential constructs, particularly emptiness and presence of meaning, in school-based screening and prevention efforts, pending longitudinal validation of their predictive utility.

## Data Availability

The raw data supporting the conclusions of this article will be made available by the authors, without undue reservation.
